# HIV-1 gp120 Interactions with Nicotine Modulate Mitochondrial Network Properties and Amyloid Release in Microglia

**DOI:** 10.1007/s11064-025-04357-3

**Published:** 2025-02-24

**Authors:** Alexandru Graur, Natalie Erickson, Patricia Sinclair, Aya Nusir, Nadine Kabbani

**Affiliations:** 1 School of Systems Biology, George Mason University, Fairfax, VA 22030 USA; 2 Interdiscplinary Program in Neuroscience, George Mason University, 4400 University Drive, Fairfax, VA 22030 USA; 3 4400 University Drive, Fairfax, VA 22030 USA

**Keywords:** Nicotinic receptor, APP, Proteomics, Autophagy, Amyloid plaques

## Abstract

Human immunodeficiency virus (HIV) infections remain a significant public health burden globally with infected individuals at high risk for cognitive decline and memory loss even on combination antiretroviral therapy. Almost half of HIV infected individuals smoke, which drives poorer health outcomes including a higher dementia rate. Microglia are the brain’s immune cells that serve as a persistent HIV reservoir contributing to neuroinflammatory signaling. We examined interactions between the HIV envelope glycoprotein gp120 and nicotine within human microglia cells (HMC3) that endogenously express chemokine receptor 5 (CCR5) and nicotinic acetylcholine receptors (nAChRs). Liquid chromatography coupled to electrospray ionization mass spectrometry (LC-ESI/MS) shows that gp120 alters mitochondria proteins within HMC3 cells. In the presence of nicotine, gp120 increased the expression of mitochondrial prohibitin 2 (PHB2), cytochrome c (cyt c), and mitofusin 2 (MFN2) but decreased fission 1 (FIS1) levels. An analysis of mito-YFP expression confirms that interaction between nicotine and gp120 increases the size and branching of mitochondrial networks. Interaction between nicotine and gp120 is also surprisingly found to promote the release of amyloid precursor protein (APP) peptides from microglia. This was accompanied by visualization of amyloid containing vesicles that colocalized with the autophagy protein LC3B-II in the cell. Taken together, our findings show that interaction between nicotine and gp120 impact microglia in a manner that regulates mitochondrial proteins and network properties and impacts amyloid protein management and release within microglia. These mechanisms may contribute to understanding neuroinflammatory signaling in smokers with HIV.

**Supplementary Information:**

The online version contains supplementary material available at 10.1007/s11064-025-04357-3.

## Introduction

The human immunodeficiency virus type 1 (HIV-1) is a retrovirus responsible for most HIV infections globally [1, 2]. HIV can integrate into the genome of its host causing long-term immunological suppression that culminates in acquired immunodeficiency syndrome (AIDS) if not treated [3]. Developments in combination antiretroviral therapy (cART) have minimized the occurrence of AIDS in much of the developed world however HIV infections are on the rise globally and particularly within sub-Saharan Africa [4]. cART managed HIV still presents significant health risks, particularly when accompanied by comorbidities such as co-infections and chronic conditions like hypertension and diabetes [5,6]. Smoking also poses a significant health risk to individuals with HIV and ~ 50% of HIV infected individuals smoke tobacco products [6, 7]. The use of tobacco products is shown to increase the incidence of lung cancer and cardiovascular disease in HIV infected individuals [8]. In addition, tobacco product use is reported to exacerbate HIV associated neurocognitive disorders (HAND) [9, 10].

HAND encompasses a spectrum of cognitive dysfunction including memory loss, irritability, and depression that may stem from virus related synaptic dysfunction, neuronal death, and enhanced inflammation within cortical and subcortical regions [11, 12]. Because HIV-1 does not appear to infect neurons, HAND is driven by the effect of HIV within glia [13, 14]. Microglia, the immune cells of the brain, have been shown to contribute to HAND through cytokine and chemokine release as well as modified phagocytic activity [15]. Studies indicate that microglia can also participate in the development of amyloid plaques within the brains of HIV infected individuals [16].

Several HIV proteins have been shown to contribute to the mechanisms of HAND including the viral envelope glycoprotein gp120. Gp120 mediates interaction of the virus with the host cell through binding to chemokine receptor CD4, CCR5, and CXCR4 [17]. Studies indicate that gp120 can also bind to nicotinic acetylcholine receptor (nAChR) through a 3 finger toxin structure found in nAChR binding proteins such as bungarotoxin and the rabies virus glycoprotein (RBVG) [18]. Interactions between gp120 and nicotine are shown to impact neuronal activity amplifying neurotoxic outcomes in various experimental systems [18]. In this study we tested the effect of nicotine on gp120 mediated signaling in microglia. We identify an interactive effect of nicotine and gp120 in microglia focused on mitochondrial regulation and amyloid release.

## Methods

### Cell Culture, Drug Treatment, and Transfection

Human microglial HMC3 cells (ATCC CRL-3304) were propagated in T75 flasks with DMEM (Gibco 11995065) supplemented with 10% fetal bovine serum (FBS) and 1% pen/strep at 37 °C with 5% CO2. This cell line is not listed as a commonly misidentified cell line by the International Cell Line Authentication Committee (ICLAC). All experiments were conducted on cells that did not exceed 25 passages. Cells were plated onto a 100 µg/ml poly-D-Lysine (Millipore A-003-E) coated matrix 24 h prior to any treatment Cell viability was measured using trypan blue staining method [19]. For proteomic analysis, cells were grown to 70% confluence then treated with 500 pM gp120B dissolved in PBS (catalog #12570) or gp120C dissolved in PBS (catalog #12582) obtained from the National Institutes of Health (NIH) AIDS Reagent Program. In experiments involving nicotine, cells were pre-treated with 10 µM nicotine dissolved in PBS (Sigma-Aldrich N3876) for 48 h then 500 pM gp120 was added to the nicotine treatment media for an additional 24 h. Media was changed daily and compounds replenished. Release assays were performed from cells grown in serum free DMEM for 24 h. Lipopolysaccharide, 100 pM (LPS) dissolved in DMEM media (Sigma-Aldrich L2630) was added 2 h before media collection under conditions of stimulated release. In experiments quantifying amyloid proteins and LC3B, autophagic flux was blocked by application of 20 nM of bafilomycin A1 (Caymanchem 11038) during 24 h before the end of the experiment. Mitochondrial dynamics were measured in cells transfected with mitochondrial matrix targeted EYFP (pCAG mitoYFP, Addgene plasmid # 168508, Lewis et al., 2016) using Lipofectamine 2000 (Thermo Fisher 11668030) [20, 21]. cDNA constructs were propagated in DH5α cells (Thermo Fisher 18258012) and purified using the Qiagen plasmid maxi kit (12162).

### Protein Extraction and Western Blot

Proteins were extracted from cultured cells as previously described [22]. Briefly, cells were lysed using a 0.1% Triton lysis buffer (Triton X-100, 150 mM NaCl, 20 mM Tris HCl, 2 mM EDTA, and 10% glycerol, supplemented with protease inhibitors (Roche, Complete Mini 11836153001) and PhosSTOP (Sigma Aldrich 4906845001)). Protein concentration was measured using the Bradford assay. Following separation on a NuPAGE 4–12% Bis-Tris gradient gel (Thermo Fisher NP0322BOX), proteins were transferred onto a nitrocellulose membrane (Thermo Fisher IB301002). Membranes were blocked with 2–5% milk before applying primary antibodies: LC3B (1:1000, Cell Signaling 2775), MFN2 (1:5000, Proteintech 12186-1-AP), FIS1 (1:2000, Proteintech 66635-1-Ig), PHB2 (1:2000, Proteintech 12295-1-AP) cytochrome c (1:1000, AbCam ab90529) and β-actin (1:2000, Cell Signaling 3700). Horseradish peroxidase conjugated secondary antibodies were obtained from Jackson Immunoresearch (West Grove, PA, USA). SeeBlue Plus2 (Thermo Fisher LC5925) was used as a molecular weight marker. Bands were visualized using SuperSignal West Pico PLUS (Thermo Fisher 34577) via the G: BOX Imaging System and GeneSYS software (Syngene, Frederick, MD, USA). Band density was analyzed using Image J (NIH, Bethesda, MD, USA) and normalized to β-actin. Average band intensity measures with ± standard error of the mean (SEM) is presented from at least three separate experiments (*n* ≥ 3). Statistical significance was identified using the one-tailed Student’s t-test. Western blot results represent average values obtained from 3 independent biological experiments.

### Liquid-Chromatography Electrospray Ionization Mass Spectrometry

Proteomic analysis was conducted in data-dependent acquisition (DDA) mode as previously described [22–24]. For protein precipitation, samples were treated with acetone on ice for 5 min then centrifuged (16,000× g for 3 min). The resulting protein pellet was denatured, reduced, and alkylated using 8 M urea, 1 M dithiothreitol, and 0.5 M iodoacetamide. Proteins were digested with trypsin (0.5 µg/µl) in 500 nM ammonium bicarbonate and incubated at 37 °C for 5 h. The samples were desalted using C-18 ZipTips (Millipore), dehydrated in a SpeedVac for 18 min, and reconstituted in 0.1% formic acid.

LC-ESI MS/MS analysis was carried out using an Exploris Orbitrap 480 coupled with an EASY-nLC 1200 HPLC system (Thermo Fisher Scientific, Waltham, MA, USA). Peptides were separated on a reverse-phase PepMap RSLC C18 LC column (75 μm i.d., 15 cm length, 2 μm particle size) from Thermo Fisher Scientific, and eluted with 80% acetonitrile and 0.1% formic acid at a flow rate of 300 nl/min. A full scan at 60,000 resolving power from 300 m/z to 1200 m/z was performed, followed by peptide fragmentation using high-energy collision dissociation (HCD) with a normalized collision energy of 28%. Monoisotopic precursor selection, internal mass calibration, and 20-second dynamic exclusion were enabled through EASY-IC filters. Data was collected on peptide precursor ions with charge states ranging from + 2 to + 4. Each sample was run in three technical replicates. Proteins included in the analysis had a quantifiable spectra signal in at least 2 out of 3 technical replicates.

### Proteomic Quantification and Analysis

The SEQUEST HT search engine within Proteome Discoverer v2.4 (Thermo Fisher Scientific, Waltham, MA, USA) was used to identify proteins by matching raw MS peptide spectra against the NCBI 2018 human protein database. The search parameters were set with a mass tolerance of 2 ppm for precursor ions and 0.05 Da for fragment ions, and a false discovery rate (FDR) cut-off of 1% for peptide spectrum matches (PSM). For quantification, peptide abundance ratios were calculated using precursor ion quantification in Proteome Discoverer, with the vehicle control group as the denominator. Statistically significant abundance ratios (adjusted p-values < 0.05) were identified using a Student’s t-test. Clustering analysis was performed using the Markov Cluster Algorithm (MCL) with an inflation parameter of 3 in the Search Tool for the Retrieval of Interacting Genes/Proteins (STRING) database [25]. Data analysis, organization, and presentation were conducted using the R packages (R Core Team, 2021): ggplot2 [26], tidyverse, Excel, GeneCards [27] and DAVID [28, 29]. For amyloid peptide release, direct PSM counts were used for comparing amyloid precursor protein (APP) across experimental conditions with 200 ng of lysozyme (Sigma Aldrich 12650-88-3) added as an internal standard. Proteomic studies are based on 3 biological repeats of the same experiment.

### Immunocytochemistry and Cell Imaging

HMC3 cells were fixed in a solution of 1x PEM (80 mM PIPES, 5 mM EDTA, and 1mM MgCl2, pH 6.8) and 0.3% glutaraldehyde, then quenched with 2 mg/ml sodium borohydride. Cells were permeabilized with 0.05% Triton X-100 (Sigma Aldrich). Images were taken with an inverted Zeiss LSM800 confocal microscope using the Zen software package (Carl Zeiss AG, Oberkochen, Germany). Cell image analysis was conducted using confocal z-stack imaging at 0.7 μm segment distances from the top to the bottom of the cell. Immunolabeled 6E10 (1:1000, BioLegend 803001) vesicles were manually quantified based on a size inclusion criterion (1.5–5 μm diameter) and visualization of a full vesicle border. Statistical analysis was conducted using a Student’s two tailed t-test. For mitochondrial network analysis, mitochondrial parameters were measured using ImageJ (NIH, Bethesda, MD, USA). Mitochondrial network analysis was conducted using an ImageJ/Fiji Mitochondria Analyzer plugin of the mito-YFP signal essentially as described in Chaudhry et al., 2020. Statistical analysis of mitochondrial parameters was conducted using Welch’s two tailed t-test. Imaging experiments were conducted on 3 independent biological replicates.

### Data Availability

Proteomic data is uploaded in the online open access Figshare repository (10.6084/m9.figshare.26116714.v1).

## Results

### Identification of gp120 Associated Proteomes in Human Microglial Cells

**Fig. 1:**
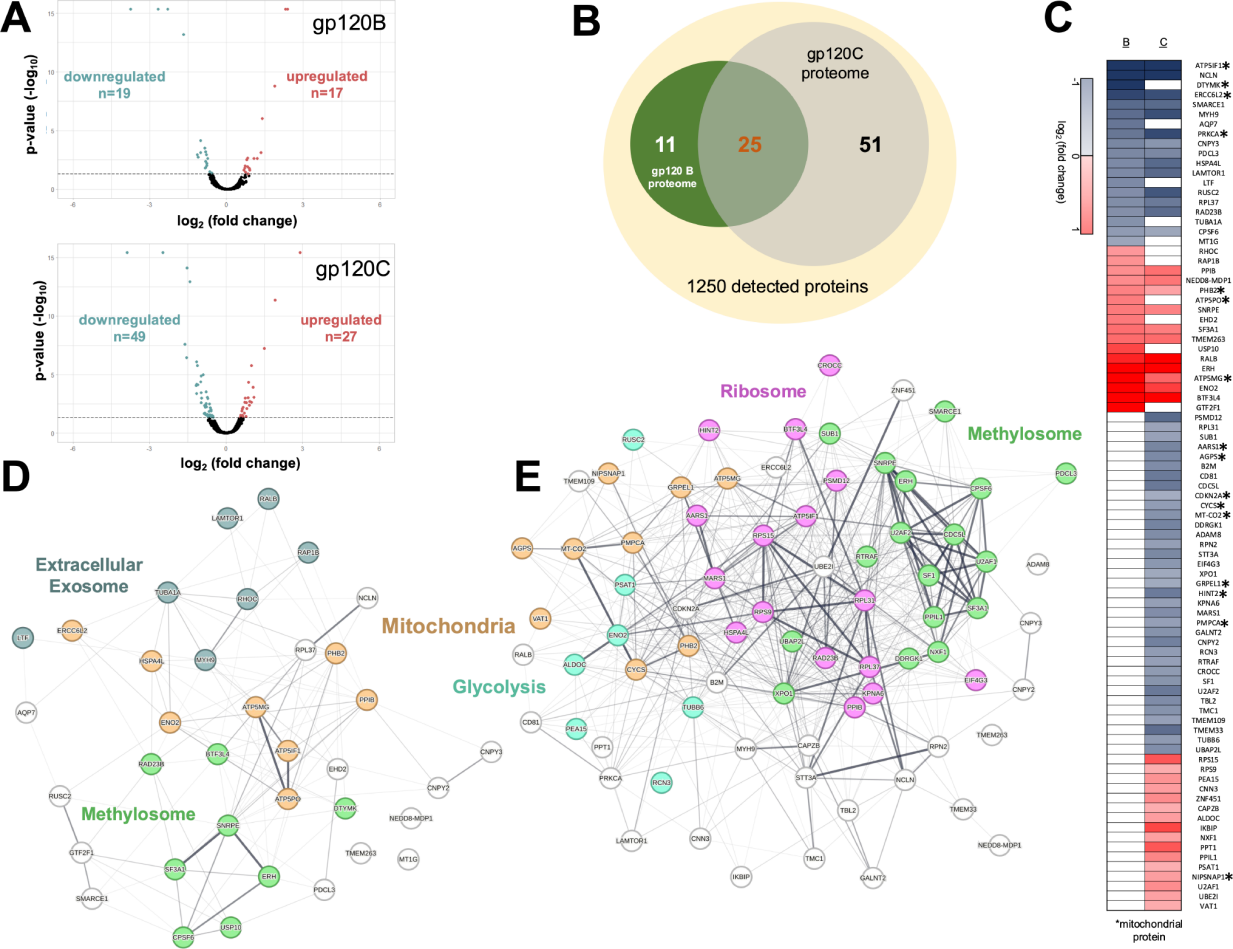
Proteomic analysis of gp120B and gp120C signaling. **A**) Volcano plots showing the distribution of detected proteins within cells. Threshold for statistical significance is *p* < 0.05. **B**) A Venn diagram illustrating the relationship between gp120B and gp120C proteomes. **C**) A heatmap showing the abundance ratio of altered proteins. Asterisks indicate mitochondrial proteins. **D**-**E**) STRING analysis of gp120B (**D**) and gp120C (**E**) proteomes. Line thickness reflects the confidence between node associations. MCL was used to identify functional color-coded clusters within the proteome network

HIV-gp120 has been shown to have widespread effect in the brain causing damage to neurons as well as driving reactivity in astrocytes and microglia [31]. To determine the effect of gp120 in microglia, we used the human microglia cell line (HMC3) that is shown to express the gp120 receptor CCR5 [32] as well as several nAChRs [33]. HMC3 cells were exposed to gp120 B or gp120 C at 500pM concentration for 24 h [34]. These treatment conditions were not associated with cellular toxicity as determined by a trypan blue exclusion assay (Supplement Fig. 1). Whole cell proteomic analysis was conducted using label-free LC-ESI MS analysis similar to earlier studies [22–24]. We identified a total of 1250 proteins across the experiment. Proteins and raw data are publicly available (10.6084/m9.figshare.26116714.v1). Statistical analysis of the protein abundance ratios was used to compare differences in individual proteins between the experimental condition (i.e., gp120 treated) and the vehicle treated control. This analysis showed that gp120B is associated with a significant change in 36 cellular proteins while gp120C was associated with a significant change in 76 cellular proteins (*p* < 0.05). These altered proteins are thus presented as the “proteome” for each of the gp120 variants. Volcano plots show the distribution of gp120B and C proteomes (Fig. 1A). 70% (25 proteins) of the gp120B proteome was found within the gp120C proteome (Fig. 1B). A heatmap chart shows the direction of change in gp120 B and C proteins (relative to the control). Shared proteins within the gp120B and C proteomes show increases/decreases in the same manner when compared to the control (Fig. 1C).

We conducted bioinformatic analysis on both proteomes using MCL analysis to define protein-protein interaction (PPI) networks important for cell function. MCL analysis is represented by an integrated STRING PPI network based on the identity of the significantly altered proteins within gp120B and C datasets (Fig. 1D-E). Within the gp120B PPI network, we detected 3 main clusters: extracellular exosome, methylosome, and mitochondria (Fig. 1D). Within the gp120C PPI network, we detected 4 clusters: ribosome, methylosome, mitochondria, and glycolysis (Fig. 1E). Notably both methylosome and mitochondria appeared within both PPI networks. Similar mitochondrial proteins were also represented across the two PPI networks. These mitochondrial proteins are marked within the heatmap (Fig. 1C). We used the human MitoCarta v3.0 database [35] to classify (pathways and sub-compartments) for these mitochondrial proteins. As shown in Table 1, gp120 proteomes include proteins important for oxidative phosphorylation (OXPHOS) (e.g. ATP5 subunits), mitophagy/autophagy (e.g. NIPSNAP-1), and the regulation of protein homeostasis (e.g. prohibitin (PHB2)). Within the gp120C cluster, several glycolysis pathway proteins were increased. These proteins include γ- Enolase (ENO2) and Aldolase (ALDOC) and appear to be connected to the mitochondrial cluster through metabolic activity [36].

**Table 1:**
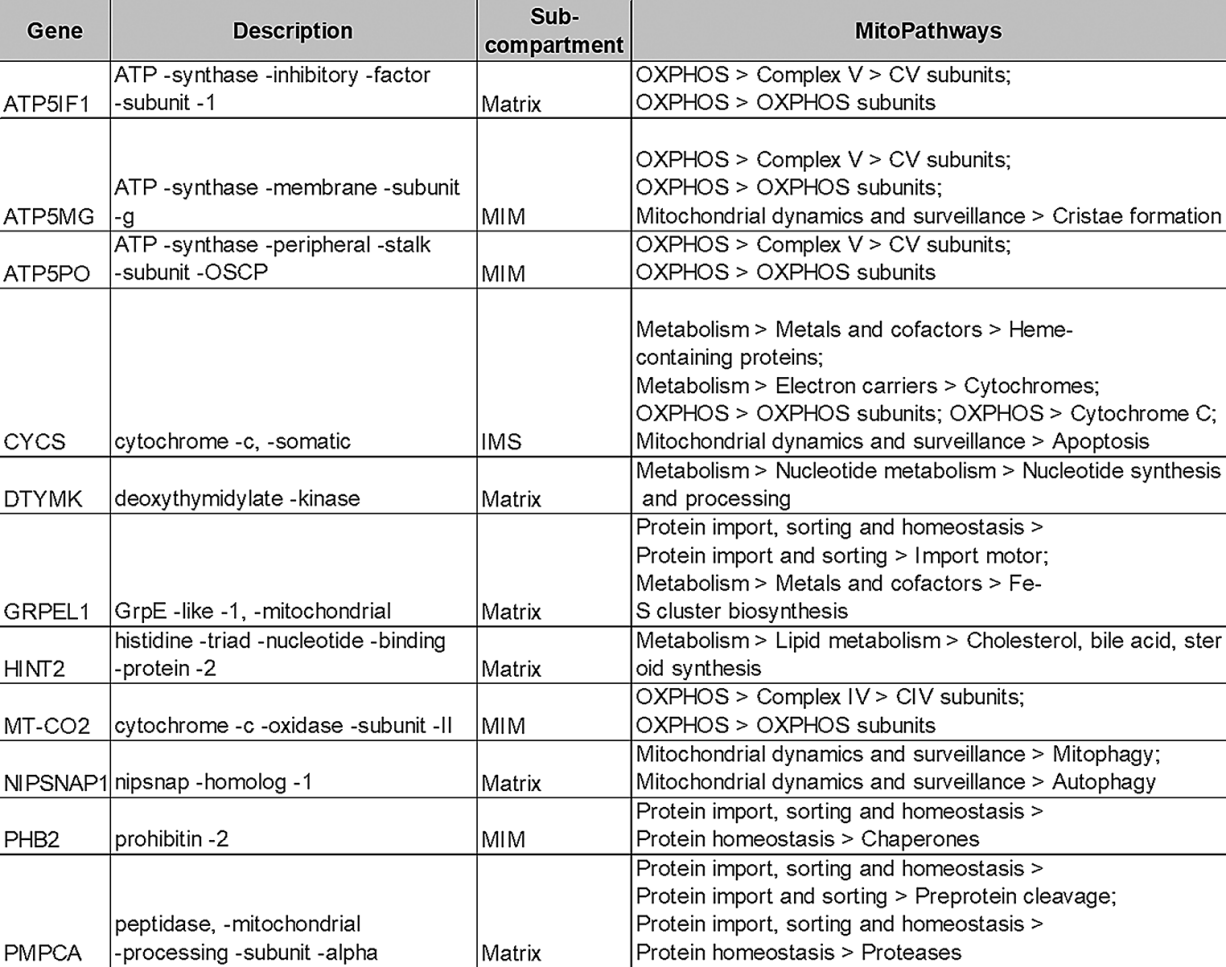
Human mitocarta v3.0 classification of mitochondrial proteins within the gp120 proteome

### An Effect of gp120c and Nicotine on Mitochondrial Proteins and Dynamics

**Fig. 2:**
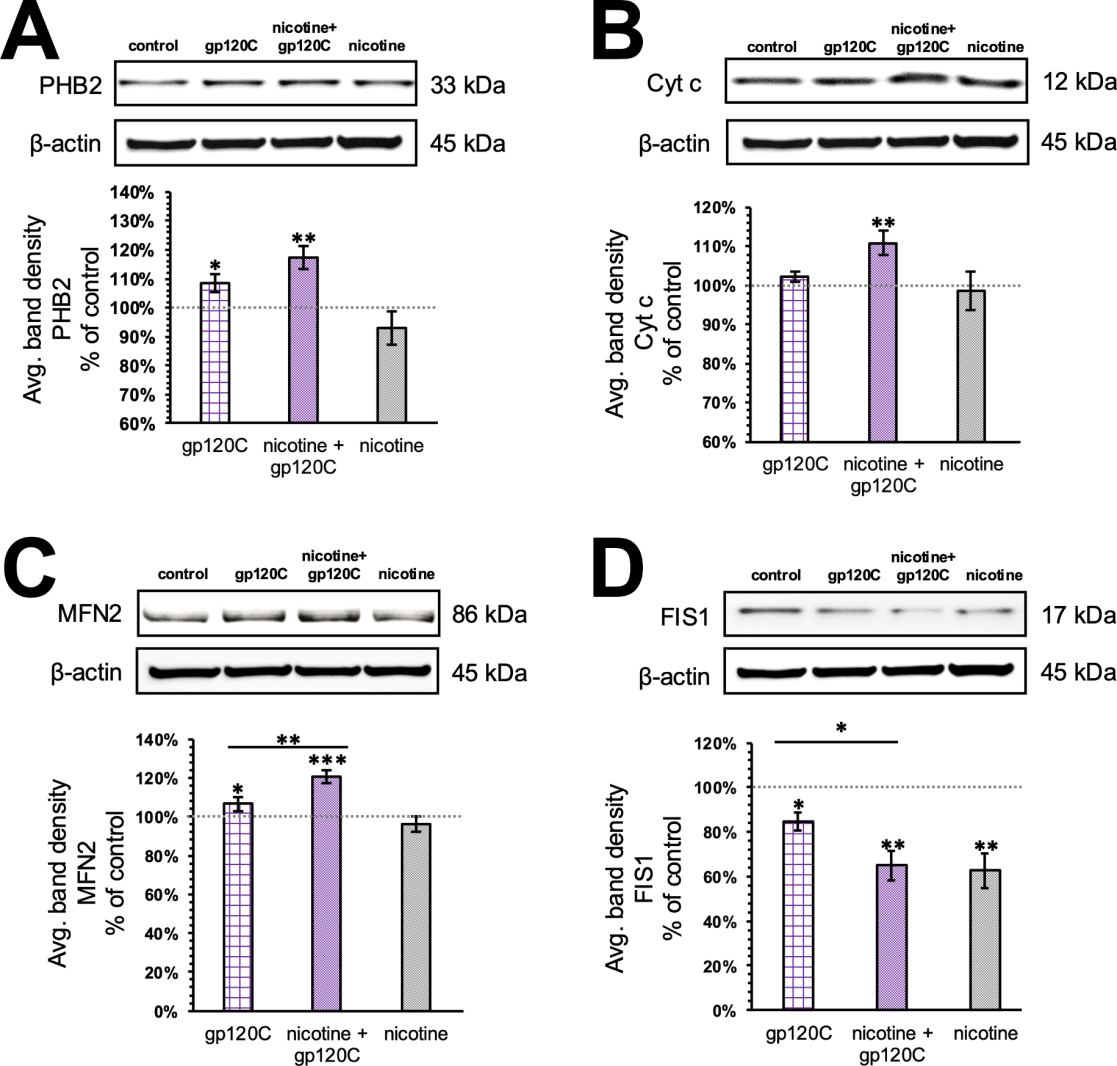
Pharmacological interaction between nicotine and gp120C modify expression of mitochondrial proteins. **A**-**D**) Top, representative immunoblots showing the effect of the control, gp120C (alone), nicotine with gp120C, and nicotine (alone) on protein expression. Bottom, histograms showing average band density (± SEM) from ≥ 3 independent experiments. * *p* < 0.05, ** *p* < 0.01, *** *p* < 0.001, Student’s t-test

We identified PHB2 as a significantly increased protein in response to gp120B and gp120C. In previous studies, we have shown that both PHB1 and PHB2 are modified by amyloid beta 1–42 (Ab42) treatment and mitochondrial neurotoxicity in neural cells [24]. To confirm changes in PHB2 expression within HMC3 cells, we used western blot detection. Cells were treated with gp120C in the same manner as the proteomic assay (24 h at 500pM). As shown in Fig. 2A, PHB2 expression was found to be significantly increased (by 8%) with gp120C treatment relative to the control. To examine interaction between nicotine and gp120C, we conducted similar experiments in HMC3 cells that were treated with 10µM nicotine for 48 h and then gp120C co-application with nicotine for another 24 h. This treatment timeline allows us to assess the impact of gp120 and nicotine within a single cell culture passage based on an average cell doubling time of ~ 2–3 days [32]. The addition of nicotine and gp120 at these concentrations was not associated with cellular toxicity as confirmed by a trypan blue exclusion assay (Supplement Fig. 1). Co-treatment with nicotine and gp120C was found to significantly increase PHB2 levels (by 17%). Statistical analysis was used to compare drug effects across multiple groups. When comparing the effect of gp120C alone to gp120C with nicotine, our results show that the addition of nicotine does not significantly impact PHB2 level (*p* = 0.07). Changes in cellular cytochrome c (cyt c) levels were also detected within the gp120C proteome. Cyt c is typically localized to the inner mitochondrial membrane where it participates in electron transport chain (ETC) activity [37]. An increase in the release of cyt c into the cytoplasm however can signal inflammation and may cause apoptosis in neural cells [38]. We confirmed the effect of gp120 on cyt c level using Western blots. As shown in Fig. 2B, cyt c levels did not appear to change following gp120 treatment, an effect that contrasts with the MS finding. However, the addition of nicotine with gp120C was found to significantly increase the expression of cyt c (by 11%), revealing an interaction between nicotine and gp120 on cyt c production in microglia.

PHB2 contributes to various mitochondrial function including mitophagy, fission, and fusion [39, 40]. We assessed for the effect of gp120C and nicotine on fission (FIS1) and fusion (mitofusin 2 (MFN2)) proteins. As shown in Fig. 2C, treatment of HMC3 cells with gp120C was associated with a significant increase in MFN2 expression (by 7%). In cells co-treated with nicotine and gp120C MFN2 levels were significantly increased, as well by 21% (*p* < 0.01) (Fig. 2C). In this study, gp120C treatment was found to significantly reduce FIS1 expression relative to control by 14% (Fig. 2D). The addition of nicotine to gp120C was found to further reduce FIS1 expression (by 30%), and this effect of nicotine on gp120C was found to be statistically significant relative to gp120C alone (*p* < 0.05) (Fig. 2D). These findings indicate that gp120C promotes an increase in mitochondrial fusion and a reduction in fission. The presence of nicotine is found to exacerbate the effect of gp120 on mitochondrial dynamics.

**Fig. 3:**
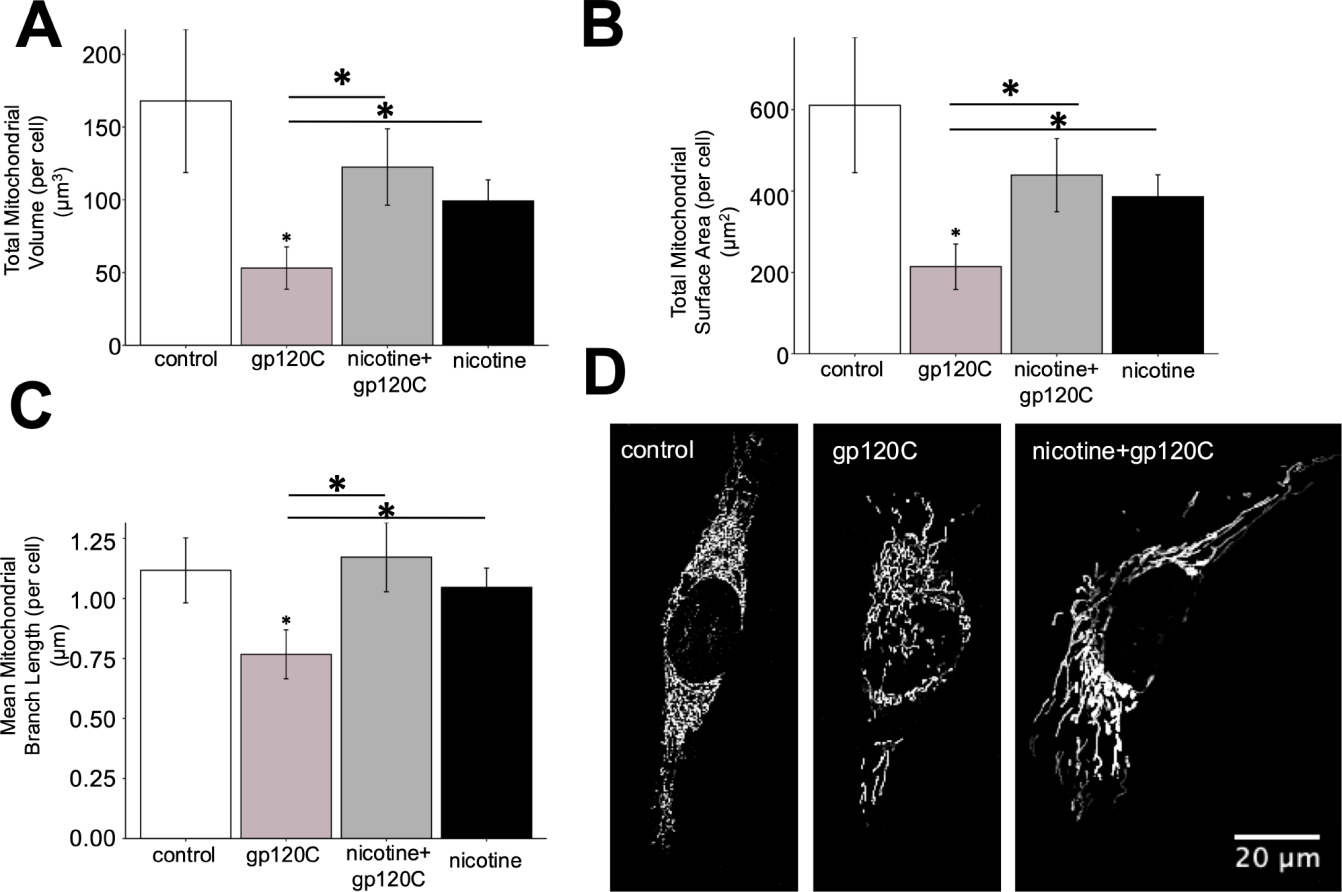
Nicotine and gp120C modify the size and shape of mitochondrial networks in microglia. **A**-**C**) Histograms showing average values (± SEM) of mitochondrial volume, surface area, and branch length. *n* = 25 cells. * *p* < 0.05, Welch’s t-test. **D**) Representative images of mitochondrial networks in HMC3 cells visualized with mito-YFP

Mitochondrion organize as networks that display temporal and spatial coordination based on motility as well as cycles of fission and fusion [41, 42]. Our earlier findings indicate an effect of gp120 and nicotine on mitochondrial fission and fusion events. We assessed changes in the structure of mitochondrial networks using confocal microscopy imaging of mito-YFP fluorescence in HMC3 cells [21]. Mitochondrial network parameters were quantified using MitoAnalyzer as described previously [30]. As shown in Fig. 3, treatment of HMC3 cells with gp120C was associated with a significant reduction in total mitochondrial volume by 69% and surface area by 65% as well branch length by 31% relative to the control. The mitoYFP signal showed a fragmented structure characterized by shorter signal length. The addition of nicotine with gp120C was found to increase mitochondrial volume, surface area, and branch length rendering mitochondrial network shapes that are more continuous. Statistical analysis indicates that nicotine and gp120C co-application is associated with an enhancement of the mitochondrial network size (volume and surface area) when compared to gp120 alone (*p* < 0.05) (Fig. 3).

### Nicotine and gp120 Regulate Amyloid Proteins

**Fig. 4:**
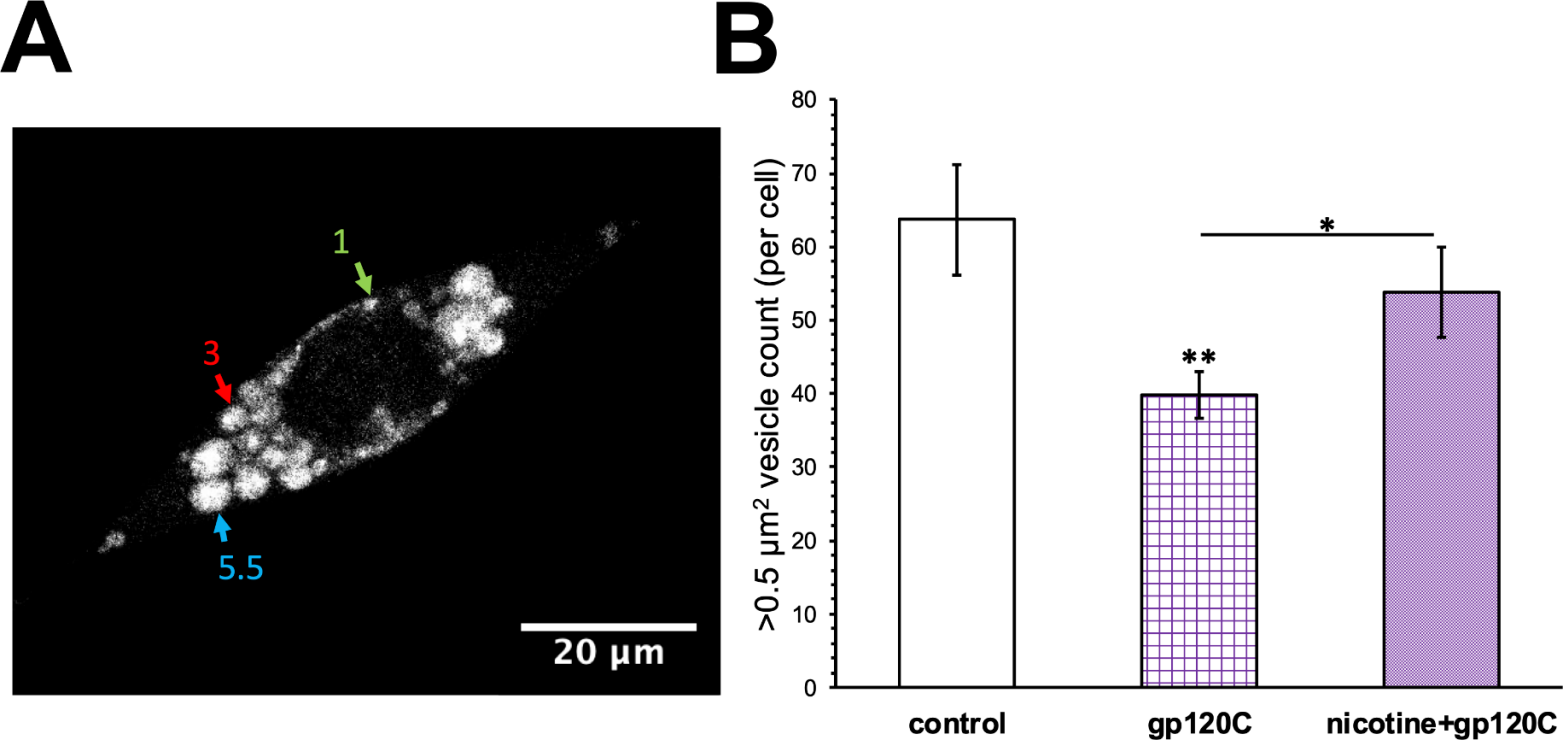
An effect of nicotine and gp120C on amyloid protein containing cellular vesicles. **A**) Representative image an HMC3 cell immunolabeled with anti-APP (6E10) antibodies. Amyloid containing vesicle clusters of various diameters (color arrows in micron units) are observed. **B**) Average number of vesicle clusters (± SEM) per experimental condition. *n* = 15, *** *p* < 0.001, Student’s t-test

Studies have shown an important role for mitochondria in the regulation and management of amyloid proteins within neurons and glia [23, 43]. Our proteomic data suggests that gp120 can alter cellular protein homeostasis through modifications in proteins such as PHB2 (Table 1) [44]. Gp120 has also been shown to participate in amyloid processing and may contribute to amyloid plaque formation in the brain during HIV infection [45]. We tested the effect of gp120C and nicotine on amyloid protein expression within HMC3 cells. Using an antibody that recognizes amino acids 1–16 of Aβ as well as the human amyloid precursor protein (APP) (6E10) [46], we immunolabeled permeabilized HMC3 cells using 6E10. As shown in Fig. 4A, gp120C treated cells exhibited punctate antibody6 expression throughout the cytoplasm. The overall distribution of the antibody labeled clusters was dependent on treatment of cells with the autophagy inhibitor bafilomycin A1 with little clustering observed in the absence of bafilomycin A1 (data not shown). When nicotine was added with gp120C, the anti-6E10 signal showed localization to vesicles within the cytoplasm. These vesicles ranged in diameter from ~ 0.07–8 μm and displayed heterogeneity in terms of their number, shape, and antibody label intensity within HMC3 cells. Niblack thresholding was applied to the images to segment particles, followed by the Watershed segmentation for particle separation. Particles within the size range of 0.50–7.00 μm² were quantified and analyzed in FIJI.

We quantified amyloid immunolabeled vesicles in gp120C or gp120C with nicotine treated cells. Vesicles that ranged in size between 0.5 and 7 μm in diameter were counted. As shown in Fig. 4B, co-application of nicotine and gp120C is found to significantly increase the number of clusters within microglia by 35%.

**Fig. 5:**
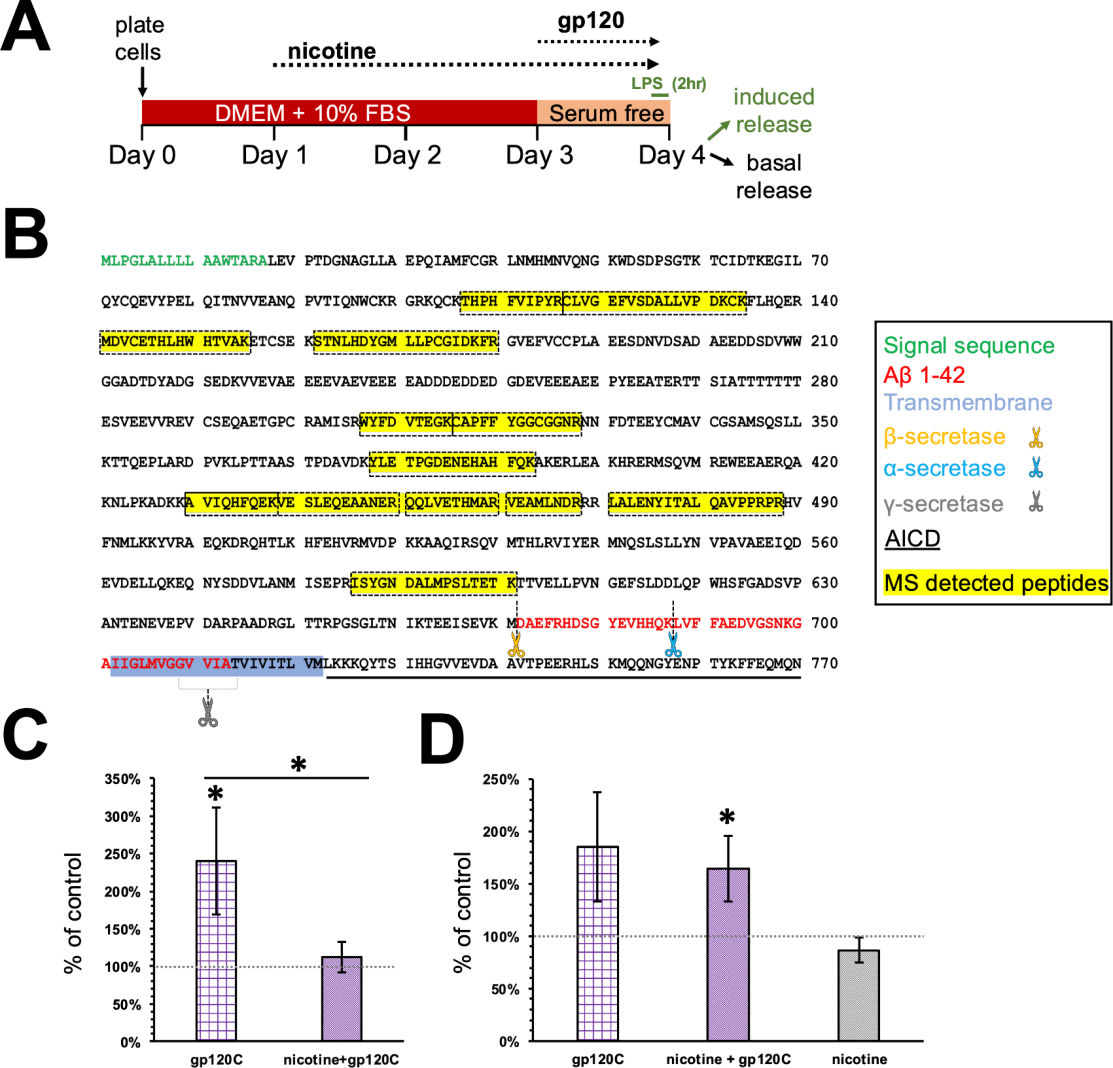
Nicotine and gp120C modulate amyloid release from microglia. **A**) Summary of the experimental design showing the time course of application for nicotine, gp120, and LPS prior to the collection of media. **B**) Amino acid sequence of human APP (UniProt; P05067) annotated to show peptide fragments identified by MS analysis (yellow) and key functional domains (AICD: intracellular domain). **C**-**D**) Average PSM count values (± SEM) for human APP peptides detected by MS analysis from supernatants of cells under basal (**C**) and LPS induced (**D**) release conditions. *n* = 3 * *p* < 0.05, Student’s t-test

Aβ peptides are formed from peptidase cleavage of membrane spanning APP in many cell types including human microglia [47, 48]. We examined the effect of gp120C or gp120C with nicotine in amyloid peptide release. Amyloid peptide release was assessed using targeted LC-ESI MS/MS from the collected extracellular media of HMC3 cells under basal or LPS stimulated conditions (Fig. 5A). We identified 13 unique peptide fragments that matched the sequence of human APP within NCBI (Fig. 5B). PSM analysis of these peptides indicates that gp120C is associated with a significant increase in amyloid peptide release by 141%. The combination of nicotine and gp120C was found to significantly decrease amyloid peptide release by 128% (Fig. 5C). LPS stimulation used to model the inflammatory state showed that amyloid peptide release was increased under gp120C, as well as gp120C and nicotine conditions (Fig. 5D). However, gp120C alone was not significantly associated with amyloid release relative to the control (*p* = 0.067) but with variability within the assay. These findings indicate an effect of gp120C and nicotine on amyloid release from microglia.

### Interaction between Nicotine and gp120 Modulates Autophagy

**Fig. 6:**
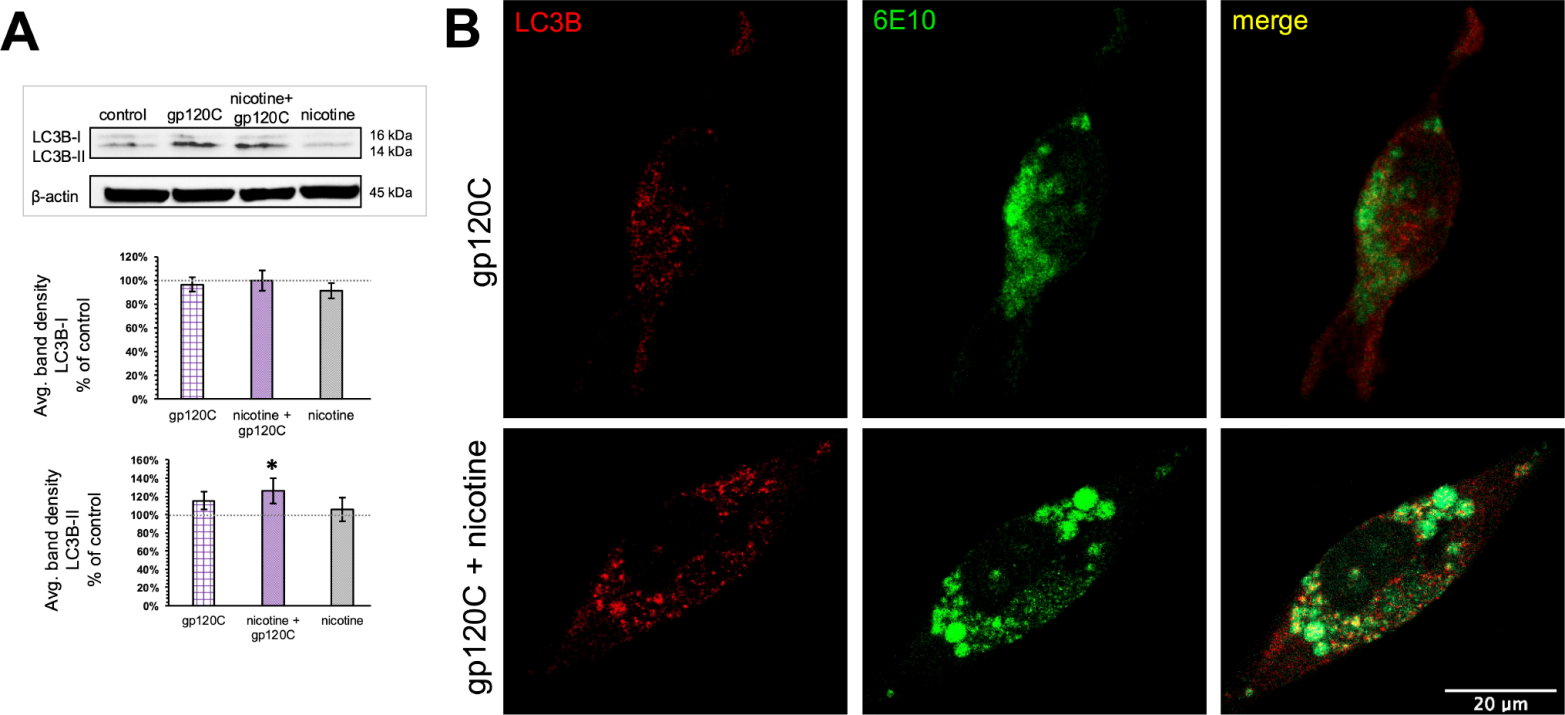
A role for nicotine and gp120C in amyloid autophagy. **A**) Top, representative blots from microglia treated with control, gp120C, and nicotine with gp120C. Bottom, average band density (± SEM) of LC3B (I and II) from the same experimental groups. *n* = 6 * *p* < 0.05, Student’s t-test. **B**) Representative images showing LC3B and 6E10 co-expression within gp120 and gp120 with nicotine treated cells. Merge panel shows partial co-localization of the two signals

Microglia can directly regulate brain amyloid proteins through phagocytic uptake and autophagy degradation implicated in the mechanisms of neurodegenerative disease including HAND [49, 50]. Our proteomic findings indicate that gp120C increases the expression of proteins such as NIPSNAP1 that regulate mitophagy and autophagy within cells [51]. We examined the effect of gp120C as well as gp120C with nicotine on the autophagy marker LC3-B in HMC3 cells. Under increased autophagy LC3B-I is lipidated resulting in LC3B-II that can be visualized at a lower molecular weight on an SDS PAGE gel [22, 52]. We assessed changes in LC3B-II based on bafilomycin A pre-treatment as before [53]. Western blot detection of LC3B-I and LC3B-II was used to quantify autophagy across the experimental groups. As shown in Fig. 6A, the addition of nicotine and gp120C significantly increased LC3B-II expression, while treatment with gp120C or nicotine alone did not significantly increase its expression. Cell imaging using anti-LC3B and 6E10 double labeling showed some co-localization between autophagy marker and amyloid proteins (Fig. 6B). Overall, we find that a subset of amyloid protein containing vesicles colocalized within autophagosomes, and this seems to be modified between cells that are treated with gp120C alone or the combination of nicotine and gp120C.

## Discussion

### Interactions Between Nicotine and HIV

Drugs of abuse have been shown to impact viral pathogenicity often rendering users at a high risk for secondary infections and chronic disease outcomes including cardiopulmonary and immune system disorder [54]. In the case of HIV, studies show that users of opioids and high-level alcohol exhibit faster cognitive decline and a great risk for neurodisease including HAND [55]. Drugs such as alcohol can also impact mitochondrial function and cause heightened inflammation throughout the brain of HIV infected individuals [56]. Similarly, morphine has been shown to disrupt mitochondrial homeostasis in neural cells resulting in an increase in the production of ROS and pro-inflammatory cytokines [57, 58]. The interaction between drugs of abuse and HIV infection within microglia is critical for understanding heightened neuroinflammation and subsequent potential for neurodegeneration within the brain. These interrelated processes are predicted to be central for the onset of cognitive and memory deficits during HAND [11].

Rising national rates in the use of nicotine products amongst young adults is driven by expansion in the use of electronic cigarettes (e-cigs) and vaping devices, which prompts concern on harmful interactions between HIV and nicotin [59, 60]. Importantly, microglia are known reservoirs for HIV and express various nAChRs [61]. Homopentameric α7 nAChRs are directly shown to mediate the release of inflammatory cytokines in response to nicotine [62, 63]. Nicotine activation of nAChRs in microglia has also been shown to regulate synaptic activity within brain areas of memory and cognitive processes including hippocampus and cortex [64, 65]. How nicotine impacts microglia modulation of synaptic responses during an HIV infection is not entirely understood.

Studies indicate an increase in proinflammatory cytokine release from microglia in response to HIV tat and gp120 exposure in cultured cells and mouse models [66]. Gp120 mainly comes from the secretion of infected microglia and virion shedding and induces the production of TNF-α, IL-1β, IL-6, as well as ROS leading to neuroinflammation and neuronal apoptosis [65]. Nicotine has been shown to exacerbate neuroinflammation and worsen the impact of HIV [67]. Interestingly, gp120 has also been shown to directly interact with α7 nAChRs in cultured cells via a short peptide sequence with homology to a 3-finger toxin structural motif found in the RBVG and neurotoxins [68, 69]. Functional interactions between HIV and the nAChR within the human brain are likely complex and influenced by various cell intrinsic factors.

Variability within the HIV genome can impact viral spread, pathogenicity, and treatment in the general population [70]. HIV-1 (subtype) B is more prevalent in North America and Europe, while HIV-1 C is found with greater frequency in sub-Saharan Africa. HIV-1 C constitutes the globally dominant type and is associated with a higher transmission rate but potentially lower HAND presentation [71, 72]. The overall sequence of gp120B and gp120C is ~ 70% identical and both subtypes are known to activate chemokine receptors such as CD4 and CCR5 [73]. In our experiments, proteomic responses to gp120B and gp120C within HMC3 microglia exhibit some similarity including mitochondrial regulation. In HMC3 cells however the actions of gp120 are likely driven through CCR5 signaling since CD4 receptors have not been detected in these cells [32]. Thus, the proteomic effects of gp120B and gp120C likely reflect responses to CCR5 signaling, which are known to be important for neuroinflammation and neurodegenerative disease [73, 74]. Whether gp120 may also bind the nAChRs within HMC3 cells is an interesting question for future studies.

### A Focus on Mitochondrial Mechanisms of Autophagy and Amyloid Protein Regulation Within Nicotine and gp120 Responsive Microglia

Latent HIV infection is predicted to drive an underlying activation of microglia, thereby perpetuating inflammation and oxidative stress within the human brain [75]. Metabolic activity can play a role in shifting microglia between two functional states: a pro-inflammatory M1 and a non-inflammatory M2 state [76]. Specifically, the M2 state utilizes mitochondrial OXPHOS as a primary source of energy, relying on mitochondrial production of ATP based on oxygen utilization. This metabolic state supports the ability of microglia to sustain high surveillance function that is important for the maintenance synapses including the clearance of extracellular debris [77]. Studies reveal that upon activation, microglia (as well as macrophages) undergo a metabolic shift towards glycolysis that although is less energy efficient provides a rapid ATP source [76]. This shift, often referred to as “metabolic reprogramming,” is essential for supporting the increased energy demands associated with the M1 activated state and enables microglia to produce inflammatory cytokines and other mediators of immune functions [78]. Studies show that during HIV, shifts in microglial metabolism exacerbate neuroinflammation contributing to neurodegeneration [79].

**Fig. 7:**
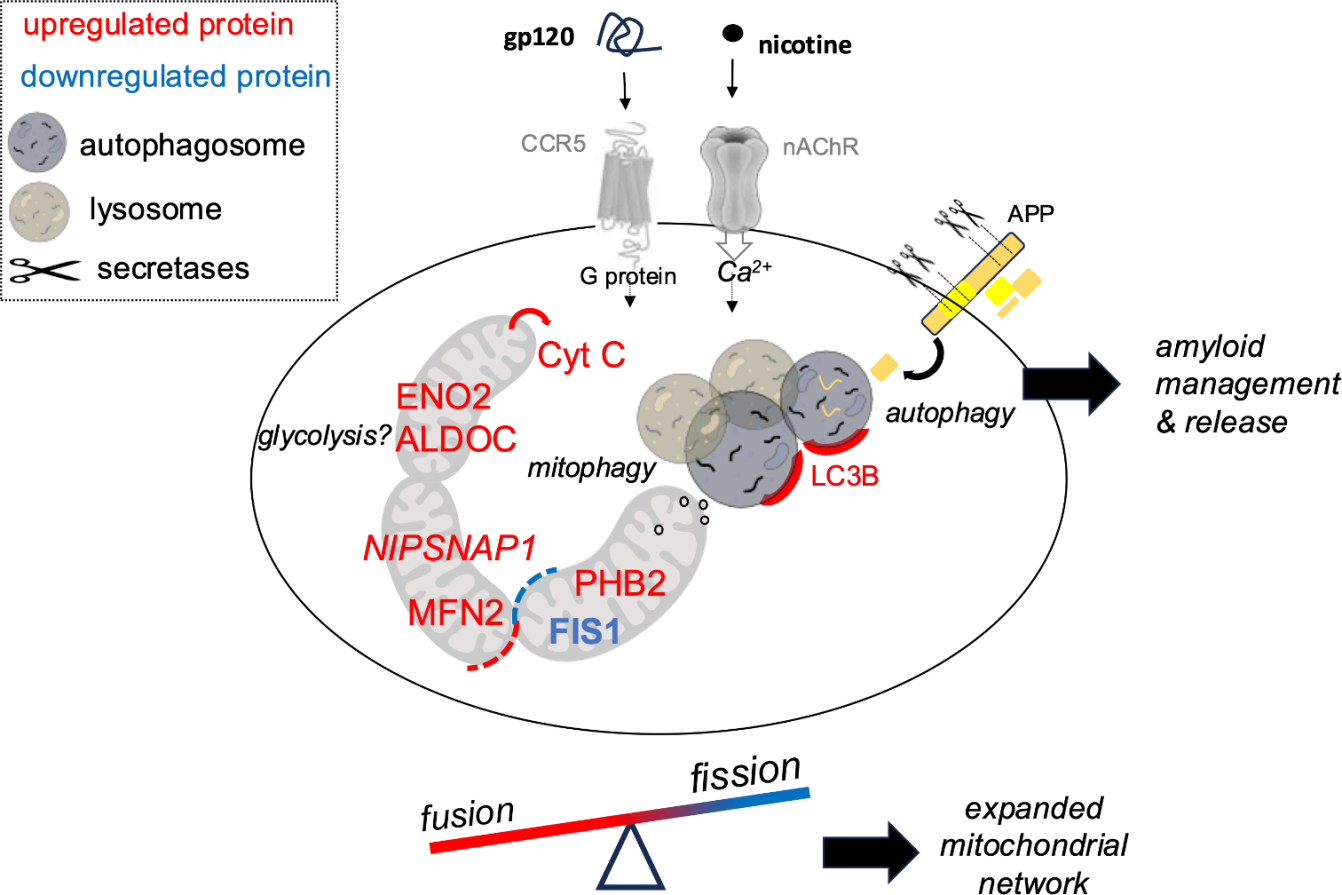
A hypothesis model showing points of interaction between nicotine and gp120 within microglia. The model highlights the effects of nicotine and gp120 on mitochondrial proteins and network properties (e.g., fission and fusion) as well as the regulation of amyloid protein (APP) processing in microglia

Our findings propose a role for interaction between nicotine and gp120 in microglia metabolic processes as suggested by modifications in proteins that influence mitochondrial network shape, functional properties, and mechanisms of autophagy and amyloid protein release (Fig. 7). At present, however we cannot conclude a specific effect for nicotine and gp120 on the M1 or M2 phenotypic state of the microglia. Future studies are needed to determine if gp120 and nicotine impact mitochondrial metabolic activity and the inflammatory status of microglial cells. Based on our proteomic findings we hypothesize an increase in glycolysis since we observed upregulation in two glycolytic proteins: ENO2 and ALDOC in the presence of gp120C. ENO2 is an enolase enzyme shown to be crucial for macrophage survival under stress conditions [80]. ENO2 is also shown to regulate Aβ induced neurodegeneration [81]. Thus, the effects of nicotine and gp120 on mitochondria including changes in fission and fusion proteins and network shape, are likely reflective of the inflammatory status and the energetic demands of microglia [82]. Similar adaptations in microglia mitochondrial properties are documented in studies on interactions between HIV and other drugs of abuse. For example, methamphetamine has been shown to increase gp120-mediated NLRP3 inflammasome activation and proinflammatory responses within rodent microglia [83]. Similarly, exposure of mouse primary microglia to the HIV associated TAT protein has been shown to impact mitochondrial membrane potential and promote accumulation of damaged mitochondria and increased mitophagy. These findings are also seen in brain tissue from HIV-1 transgenic rats and demonstrate a direct effect of TAT peptides on mitochondria within microglia [84].

Mitochondrial fission generates fragmented mitochondria, while mitochondrial fusion leads to an elongated mitochondrial network. Ongoing shifts between fission and fusion rates are highly correlated with the expression of mitochondrial proteins including Drp1 and Fis1 (fission) and Mfn1, Mfn2, and Opa1 (fusion) [85]. Previous studies in mouse brains have shown that gp120 decreases Fis1 expression [86]. Our study confirms this effect of gp120 and shows that co-application of nicotine further attenuates Fis1 levels in microglia. Cellular decrease in the expression of Fis1 suggests a reduction in mitochondrial fission thus rendering larger and more connected mitochondrial networks. In morphometric analysis of mitoYFP expression, we confirm that co-application of nicotine and gp120 is associated with a greater mitochondrial surface area and volume. Likewise, we observe that gp120 treatment increases the expression of the mitochondrial fusion protein Mfn2, and that the addition of nicotine enhances this effect. Taken together, these findings demonstrate asynergistic interaction between nicotine and gp120 on mitochondrial regulation within microglia and suggest that nicotine may heighten the neuroinflammatory properties of HIV in the brain through mitochondrial modification [87, 88].

In one scenario, changes in mitochondrial activity may participate in the progression of brain amyloid disease during an HIV infection [89]. Mitochondria associate with various amyloidogenic proteins including neurotoxic amyloids such as Aβ42 that are shown to modify mitochondrial activity and proteomes in neural cells [24, 90]. In this study, we observe that interactions between nicotine and gp120 alter the release APP peptide products, which may contribute to amyloid protein pathology [91]. It is interesting to note that smoker’s maybe at a greater risk for HAND related cognitive disease based on amyloid pathology [92]. Our experiments indicate an effect of nicotine and gp120C co-application on not only the release of amyloid proteins but also on their localization within the cell. Specifically, we observe that APP immunoreactive clusters can colocalize with the autophagosome marker (LC3B). In the absence of the autophagy inhibitor Bafilomycin A however we observe that these APP immunoreactive clusters are reduced pointing to the role protein degradation through autophagosome/lysosome interaction in amyloid protein regulation. It is interesting to consider that intralumenal vesicles can also interact within this pathway and contribute to exosome mediated amyloid release [93, 94]. This is supported by findings that either nicotine or gp120 alone can modulate exosome release [95, 96].

Proteomic assessment of the released APP peptides indicates that co-application of nicotine and gp120C alters amyloid protein release from microglia and that this process is modified by LPS activation. In recent studies, HIV has been shown to increase amyloid peptide production in microglia [87]. These findings suggest that nicotine in conjunction with HIV neurotoxic peptides such as gp120 can impact microglia activity potentially contributing to amyloid pathology in the brain.

## Electronic Supplementary Material

Below is the link to the electronic supplementary material.

Supplementary Material 1

## Data Availability

No datasets were generated or analysed during the current study.
