# Supplementary material for: HIV-1 gp120 Interactions with Nicotine Modulate Mitochondrial Network Properties and Amyloid Release in Microglia
Source: Neurochem Res. 2025 Feb 24;50(2):103. doi: 10.1007/s11064-025-04357-3 (PMC11850467; doi:10.1007/s11064-025-04357-3)
Supplement: Supplementary file 1 — Supplementary Material 1 [file 11064_2025_4357_MOESM1_ESM.pdf]

Supplement Figure 1. HMC3 cell viability assay assessed by a trypan blue exclusion assay. The percentage of living cells is compared to total cell count under the same experimental conditions: control (PBS for 72 hr), gp120C (500pM for 24 hr), nicotine (10μM for 72 hr) and nicotine+gp120C (10μM nicotine for 72 hr + 500pM gp120C for the last 24 hr). Top histogram: Average values ± standard error of the mean (SEM) from 3 separate experiments. Bottom table: Student's t-test analysis does not show an effect of an experimental condition ($p > 0.05$).

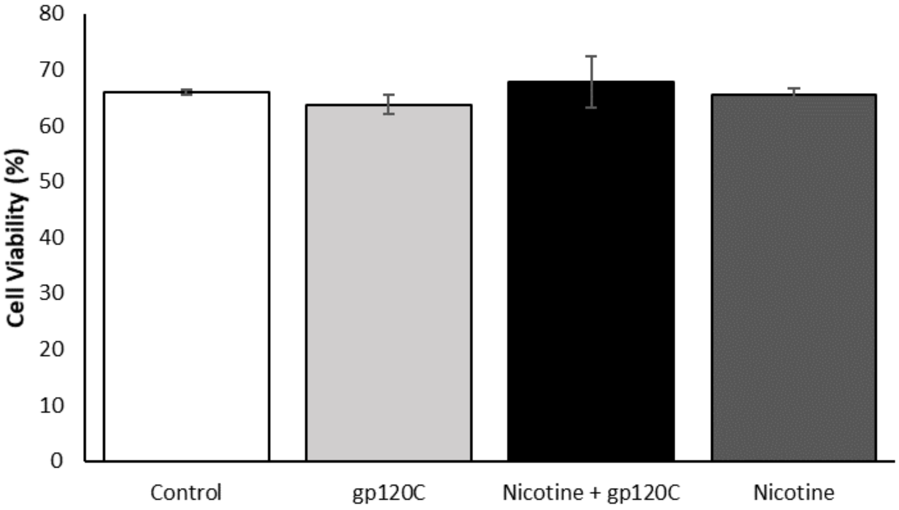

Groups	t-statistic	p-value
PBS vs Nicotine	0.368	0.7316
PBS vs Nicotine+gp120C	0.369	0.7308
PBS vs gp120C	1.245	0.2810
Nicotine vs Nicotine+gp120C	0.458	0.6711
Nicotine vs gp120C	0.850	0.4433
Nicotine+gp120C vs gp120C	0.805	0.4661
